# Parental Rejection, Overprotection and Adolescent Smartphone Addiction: Mediating Role of Sense of Security and Moderating Role of Forgiveness

**DOI:** 10.3390/bs16050796

**Published:** 2026-05-16

**Authors:** Wuyu Wang, Kairu Xue, Lu Zhou, Fanchang Kong

**Affiliations:** 1 Key Laboratory of Adolescent Cyberpsychology and Behavior (CCNU), Ministry of Education, Wuhan 430079, China; wwy2024110849@mails.ccnu.edu.cn (W.W.);; 2 School of Psychology, Central China Normal University, Wuhan 430079, China; luzhou2023@mails.ccnu.edu.cn; 3 Key Laboratory of Human Development and Mental Health of Hubei Province, Wuhan 430079, China

**Keywords:** parental rejection, parental overprotection, smartphone addiction, sense of security, forgiveness

## Abstract

This study examined a moderated mediation model linking parental rejection and overprotection to smartphone addiction, with sense of security as a mediator and forgiveness as a moderator. A total of 730 students (mean age = 12.15 ± 1.13 years; 50.7% female) were recruited from two primary and two secondary schools in Hunan, China, using cluster sampling by class, and all participants completed a set of self-report questionnaires. Results showed that, after controlling for gender and age, both parental rejection and overprotection were positively associated with smartphone addiction and negatively associated with sense of security and forgiveness. Sense of security partially mediated the links between negative parenting and smartphone addiction. Interpersonal forgiveness moderates the direct associations between parental rejection, overprotection and adolescent smartphone addiction, and self-forgiveness moderates the relationships between sense of security and smartphone addiction. The present study clarifies the associations between negative parenting behaviors (i.e., parental rejection and overprotection) and problematic smartphone use in early and middle adolescence, highlights the vital protective roles of security and forgiveness, and provides empirical evidence to inform the prevention and intervention strategies for adolescent smartphone addiction.

## 1. Introduction

Smartphones have brought tremendous convenience to adolescents’ lives, yet they also make users highly susceptible to excessive use, psychological dependence, and cravings, potentially leading to smartphone addiction (Duke & Montag, 2017). Smartphone addiction refers to a behavioral addiction in which inappropriate and excessive smartphone use results in negative physiological and psychological consequences (Leung, 2008; He et al., 2024). Studies have shown that an increasing number of adolescents are experiencing smartphone addiction. In the United Kingdom, 38.9% of individuals aged 18–30 reported symptoms of “smartphone addiction” (Sohn et al., 2021). Similarly, a survey of 2639 Chinese adolescents found that over 20% met the criteria for smartphone addiction (Zou et al., 2019). A meta-analysis of 33,831 adolescents and young adults (aged 15–35) across 24 countries indicated that problematic smartphone use increased globally between 2014 and 2020 (Olson et al., 2022). Adolescents, in particular, are a highly vulnerable group for smartphone addiction, as they are more easily attracted to smartphones (Lee & Kim, 2018) and tend to have lower awareness of internet-related risks (Lareki et al., 2017).

As the most crucial developmental context for adolescents, the family environment plays an irreplaceable role in shaping their psychological and behavioral development (Jang & Ji, 2012). Parenting is a key mechanism through which the family environment influences adolescents, encompassing parental behaviors and strategies aimed at controlling and socializing their children (Baumrind, 1966). Among these, negative parenting primarily includes parental rejection and overprotection (Vera et al., 2011). Research has demonstrated a strong association between negative parenting and adolescent smartphone addiction (Ma et al., 2021); however, few studies have explored the specific mechanisms through which negative parenting contributes to smartphone addiction. Therefore, this study aims to clarify the relationship between negative parenting and adolescent smartphone addiction, as well as the roles of potential protective factors in this process.

## 2. Literature Review

### 2.1. The Associations Between Parental Rejection, Overprotection, and Smartphone Addiction

Studies have shown that negative parenting styles increase the risk of adolescent smartphone addiction (Bae, 2015). Specifically, both parental rejection and overprotection are significantly positively correlated with problematic smartphone use among college students (Chen et al., 2021; He et al., 2024) and internet addiction among vocational students (Kong et al., 2025). According to the Conservation of Resources (COR) theory, individuals with greater resources tend to adapt better, whereas those with insufficient resources are more likely to experience adaptation difficulties (Hobfoll, 2002). Parents serve as the primary resource providers for adolescents, and when they engage in negative parenting, the support, encouragement, and affirmation they provide to their children decrease, leading to a reduction in the psychological resources available to the child (Fan et al., 2018). When adolescents do not receive sufficient parental attention and care to meet their psychological needs, they may turn to the internet as an alternative source of fulfillment. This excessive reliance on virtual compensation can increase the risk of internet addiction (D. Li et al., 2016).

Parents who exhibit rejection not only refrain from offering help but also spend little time with their children. As a result, their children are more likely to overuse smartphones as a means of seeking emotional comfort and psychological satisfaction. In this sense, rejecting parenting may contribute to adolescent smartphone addiction. In addition, overprotective parenting, characterized by strict monitoring and intrusive control, may lead to internalizing and externalizing problems in adolescents (De Roo et al., 2022); adolescents with poor development of autonomy experience more severe depression and anxiety, while those with higher levels of defiance develop externalizing substance use and delinquency. Adolescents who perceive high levels of parental rejection, interference, and distrust are more likely to develop oppositional behaviors and engage in problematic behaviors (Stoltz et al., 2012). Based on the above analysis, this study proposes the following hypotheses:

**H1a.**  *Parental rejection is positively related to adolescent smartphone addiction.*

**H1b.**  *Parental overprotection is positively related to adolescent smartphone addiction.*

### 2.2. The Role of Sense of Security in the Relationships Between Parental Rejection, Overprotection, and Adolescent Smartphone Addiction

Parental rejection and overprotection may also affect adolescents by altering their internal psychological states, even before considering the direct influence of adolescent smartphone addiction. This study introduces the variable of sense of security to examine its role in the relationship between parental rejection, overprotection, and adolescent smartphone addiction. Sense of security refers to an individual’s perception of potential physical or psychological threats and their sense of competence or helplessness in responding to such threats. Individuals experience a sense of security when they can anticipate and manage possible dangers. It primarily encompasses feelings of safety in social interactions and a sense of certainty, predictability, and control over life events (Cong & An, 2004).

According to attachment theory, parenting styles shape children’s attachment patterns and their emotional bonds with parents (Bosmans et al., 2022). The cold and rejecting parenting style prevents children from forming secure and supportive emotional connections with their parents, potentially fostering an internalized belief that the external environment is threatening and hostile. This, in turn, undermines their self-identity (Drake & Ginsburg, 2012). If parents fail to provide genuine love, children may develop a profound sense of insecurity (Rosenstein & Horowitz, 1996). Studies have shown that negative parenting styles can harm adolescents’ emotional security (Peng et al., 2020; Stronach et al., 2013), and harsh, rejecting, and neglectful parenting can reduce adolescents’ sense of security (Ojha & Singh, 1988). Therefore, parental rejection and overprotection may undermine adolescents’ sense of security.

On the other hand, the diminished sense of security increases the risk of adolescent smartphone addiction. When adolescents do not receive sufficient security from real-life parent–child interactions, they may seek alternative sources of emotional fulfillment through material objects or behavioral attachments (Flores, 2006). Smartphones, as essential tools for maintaining social relationships, are particularly likely to become targets of compensatory attachment (Konok et al., 2016). Adolescents may increasingly use smartphones to fulfill unmet psychological needs in real life (Deng et al., 2012). Moreover, adolescents’ dependence on the internet can be understood as a displacement and compensation for their attachment needs, reflecting a distorted form of attachment when normal attachment processes are disrupted or unfulfilled (X. Li & Lu, 2014). Adolescents with low security may retreat into the virtual world to seek psychological satisfaction (Zhao, 2016), thereby increasing the risk of smartphone addiction. This assumption is also supported by meta-analyses indicating that insecure attachment is a significant predictor of smartphone addiction (Zhang et al., 2022). According to Uses and Gratifications Theory, which posits that individuals use smartphones to satisfy psychological needs (Kang & Jung, 2014; Wei et al., 2024), when individuals find that their psychological needs are met through smartphone use, they are more likely to engage in excessive smartphone use, ultimately leading to addiction.

Based on the above analysis, negative parenting may reduce adolescents’ sense of security, and smartphones may serve as a substitute for fulfilling the psychological needs associated with reduced security, thereby increasing the risk of excessive smartphone use. Thus, this study proposes the following hypotheses:

**H2a.**  *Sense of security mediates the relationship between parental rejection and adolescent smartphone addiction.*

**H2b.**  *Sense of security mediates the relationship between parental overprotection and adolescent smartphone addiction.*

### 2.3. The Role of Forgiveness in the Relationships Between Parental Rejection, Overprotection, and Adolescent Smartphone Addiction

The impact of parental rejection and overprotection on adolescent smartphone addiction may vary depending on adolescents’ psychological characteristics. Forgiveness, as a positive psychological trait, refers to the process by which an individual who has been wronged relinquishes negative emotions, cognitions, and behaviors toward the offender while fostering positive emotions, cognitions, and behaviors toward them (Enright et al., 1992). In addition, Bauer et al. (1992) introduced the concept of self-forgiveness, which refers to the psychological process of an individual forgiving them for their mistakes or sins, shifting from self-hatred to self-care. The failure of self-forgiveness is positively correlated with depression and anxiety (Maltby et al., 2001).

Previous studies have highlighted the close relationship between forgiveness and various positive psychological factors. Forgiveness can help individuals recover more quickly from emotional harm (Brady et al., 2023) and rebuild healthy interpersonal relationships (Spiers, 2004). Additionally, research has shown that forgiveness helps adolescents cope with the distress caused by parental divorce (Freedman & Knupp, 2003). Some studies have also found the protective effect of forgiveness in the context of negative parenting styles on adolescents. Children’s forgiveness can regulate the association between strict parental discipline and emotional dysregulation, thereby cutting off the indirect path from strict parental discipline to adolescent internet addiction (M. Wang & Qi, 2017). According to the developmental compensatory hypothesis (Gao & Chen, 2006), individuals who encounter adverse life environments undergo a psychological compensation process as part of their normative developmental trajectory. When possessing insufficient psychological resources, adversity in life may prompt individuals to engage extensively in online activities to alleviate negative emotions (Kardefelt-Winther, 2014), while interpersonal forgiveness, as a form of psychological compensation, can effectively alleviate negative emotions and reduce the negative impact of interpersonal offense on mobile phone addiction (Du & Wang, 2025). Therefore, we propose the following hypotheses:

**H3a.**  *Interpersonal forgiveness moderates the relationship between parental rejection and adolescent smartphone addiction.*

**H3b.**  *Interpersonal forgiveness moderates the relationship between parental overprotection and adolescent smartphone addiction.*

Moreover, the Protective–Protective Model suggests that positive traits can enhance other facilitating factors and produce synergistic protective effects (Gomez & McLaren, 2006). Security can affect problem behavior through self-evaluation types of psychological variables such as self-esteem and depression (Wu et al., 2019). As a psychological trait associated with internalized emotions such as depression and anxiety (J. C. Kim et al., 2022), low levels of security are often accompanied by a psychological process of self-denial, which weakens an individual’s ability to benefit from existing security. As a positive self-regulation strategy, self-forgiveness can effectively alleviate self-denial, enable individuals to absorb and utilize the protective resource of security more fully, thereby enhancing the inhibitory effect of security on mobile phone addiction. Therefore, we propose the following hypothesis:

**H4.**  *Self-forgiveness moderates the relationship between parental overprotection and security.*

### 2.4. The Present Study

**Figure 1:**
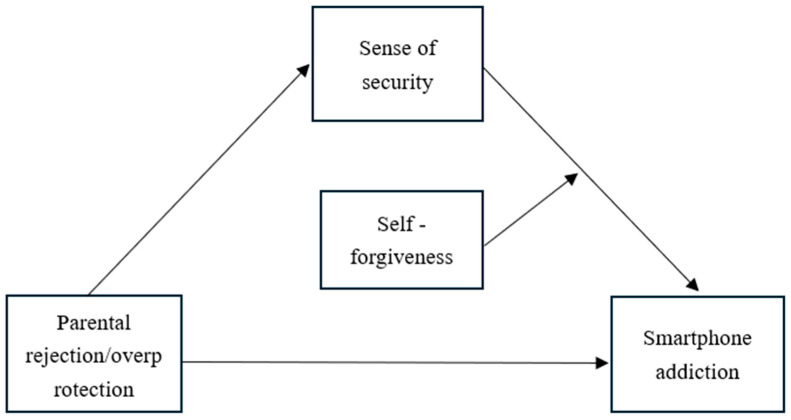
The conceptual model (Model 15 of self-forgiveness).

**Figure 2:**
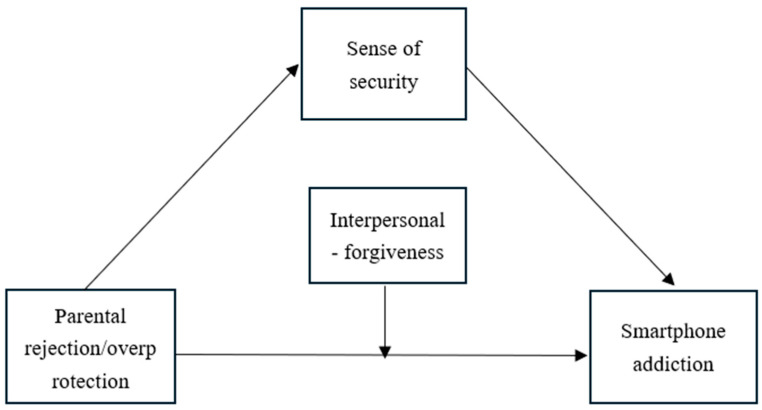
The conceptual model (Model 15 of interpersonal forgiveness).

**Figure 3:**
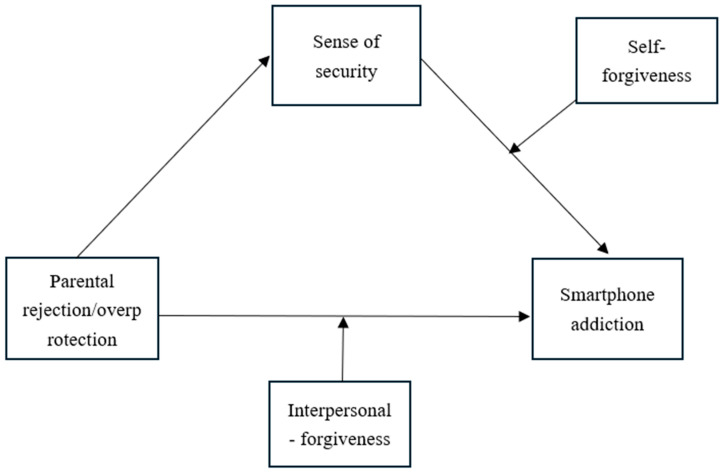
The conceptual model (Model 17).

In sum, the present study proposes a risk–mechanism–protection framework. First, drawing on Conservation of Resources theory (Hobfoll, 2002), parental rejection and overprotection are conceptualized as risk factors that deplete adolescents’ psychological resources. Second, integrating attachment theory (Bosmans et al., 2022) and the uses and gratifications theory (Kang & Jung, 2014), sense of security serves as a key mediating mechanism—negative parenting undermines security, and this insecurity drives adolescents to seek compensatory satisfaction from smartphones. Third, forgiveness operates as a protective factor with a differentiated role: based on the developmental compensation hypothesis (Gao & Chen, 2006), interpersonal forgiveness buffers the direct effects of negative parenting on smartphone addiction; based on the protective–protective model (Gomez & McLaren, 2006), self-forgiveness enhances the protective function of sense of security. The proposed models are illustrated in Figure 1, Figure 2 and Figure 3.

## 3. Method

### 3.1. Participants

A total of 730 students were recruited from two primary schools and two secondary schools in Hunan Province using cluster sampling based on class. After excluding invalid questionnaires, such as those with repetitive answers or incomplete responses, 677 valid questionnaires were obtained, yielding an effective response rate of 96.85%. Among the participants, there were 334 males (49.30%) and 343 females (50.70%). The distribution by grade was as follows: 121 fifth graders (17.90%), 234 sixth graders (34.60%), 186 seventh graders (27.50%), and 136 eighth graders (20.10%). The average age of the participants was 12.15 ± 1.13 years.

We obtained written informed consent from all participants’ parents and verbal assent from all adolescent participant. The research procedures were approved by the Ethics Committee of the corresponding author’s university.

### 3.2. Measurements

#### 3.2.1. Parental Rejection and Overprotection

The parental rejection and overprotection subscales of the shortened parenting styles questionnaire (Jiang et al., 2010) were used to assess the extent of parental rejection and overprotection experienced by adolescents. The parental rejection scale consists of 6 items, and the parental overprotection scale consists of 8 items. All items were rated on a 4-point Likert scale, from 1 (strongly disagree) to 4 (strongly agree). Higher total scores indicate stronger parental use of these parenting styles. The Cronbach’s α coefficients in this study were 0.87 and 0.73, respectively.

#### 3.2.2. Sense of Security

The security scale, developed by Cong and An (2004), consists of 16 items and includes two dimensions: Interpersonal security and certainty of control. The scale uses a 5-point Likert scale, ranging from 1 (strongly disagree) to 5 (strongly agree). Higher total scores indicate higher levels of security. The Cronbach’s α coefficient for the total scale in this study was 0.87.

#### 3.2.3. Forgiveness

Forgiveness was measured using the adolescent forgiveness tendency questionnaire developed by W. Wang et al. (2012). This questionnaire includes 22 items, covering four dimensions: revenge, forgiveness of others, self-punishment, and self-forgiveness. It uses a 7-point Likert scale, from 1 (strongly disagree) to 7 (strongly agree). Higher total scores indicate higher levels of forgiveness. The Cronbach’s α coefficient for the total scale in this study was 0.76.

#### 3.2.4. Smartphone Addiction

The phone addiction index (Leung, 2008) was used to assess the degree of smartphone addiction in individuals. This questionnaire consists of 17 items, covering four dimensions: loss of control, withdrawal, escape, and inefficiency. It uses a 5-point Likert scale, from 1 (rarely) to 5 (always). Higher total scores indicate a stronger tendency for smartphone addiction. The Cronbach’s α coefficient for the total scale in this study was 0.91.

### 3.3. Statistical Analysis

First, after excluding invalid questionnaires, missing values were minimal (less than 1% of data were missing per variable), so mean imputation was used to handle the missing data. Next, descriptive statistics and correlation analysis were conducted using SPSS 24.0. Finally, all variables were standardized, and Process Macro (Hayes, 2017) was used for moderated mediation analysis with Models 15 and 17. Given the relative independence of parental rejection and overprotection as parenting constructs, this study constructed two separate moderated mediation models to examine the roles of security and forgiveness in the relationships between parental rejection and smartphone addiction, and between parental overprotection and smartphone addiction.

Considering that interpersonal forgiveness and self-forgiveness theoretically act on different paths, we first tested the hypothesized moderating effects separately using PROCESS Model 15. Specifically, we examined (a) the moderating role of interpersonal forgiveness on the direct paths from parental rejection/overprotection to smartphone addiction, and (b) the moderating role of self-forgiveness on the indirect path from sense of security to smartphone addiction. Subsequently, to explore the unique effects of each forgiveness dimension when controlling for the other, we constructed a more saturated model (PROCESS Model 17) that simultaneously included both moderators and all relevant interaction terms. This saturated model serves as a robustness check for the specificity of the two forgiveness dimensions. In the model, Bootstrap bias-correction was used for parameter estimation (N = 5000), and 95% confidence intervals were reported to estimate the mediation effect and moderation effect. If the confidence interval does not include 0, the effect is considered significant.

## 4. Results

### 4.1. Common Method Bias

To control for common method bias, the study utilized methods such as anonymous responding and reverse scoring during data collection. Additionally, Harman’s single-factor test was conducted using exploratory factor analysis (Podsakoff et al., 2003) on all items from the four questionnaires. The results revealed that 24 factors had eigenvalues greater than 1, and the first extracted common factor explained 13.75% of the variance, which is below the threshold value of 40%. This indicates that the study does not suffer from significant common method bias.

### 4.2. Descriptive Statistics and Correlation Analysis

The mean, standard deviation, and correlation analysis results of the main variables are presented in Table 1. The correlation analysis showed that parental rejection and overprotection were significantly positively correlated with smartphone addiction, and negatively correlated with sense of security. Moreover, forgiveness was significantly positively correlated with sense of security and negatively correlated with parental rejection, overprotection, and smartphone addiction. Additionally, self-forgiveness was significantly positively correlated with inter-personal forgiveness.

### 4.3. Moderated Mediation Effect of Parental Rejection on Smartphone Addiction

The results of Model 15 (see Tables S1 and S2) show that, after controlling for gender and age, parental rejection was negatively related to adolescents’ sense of security (*β_SF_* = −0.37, *p* < 0.001; *β_IF_* = −0.37, *p* < 0.001), while sense of security was negatively related to smartphone addiction (*β_SF_* = −0.41, *p* < 0.001; *β_IF_* = −0.37, *p* < 0.001). Sense of security partially mediates the relationship between parental rejection and smartphone addiction, with a mediation effect of 0.148 and a 95% confidence interval (CI) of [0.112, 0.191], accounting for 55.04% of the total effect. The interaction of parental rejection and interpersonal forgiveness was significantly related to smartphone addiction (*β* = −0.07, *p* < 0.05), the interaction of self-forgiveness and security was significantly related to smartphone addiction (*β* = −0.08, *p* < 0.05), and the hypothesis was supported. These findings suggest that self-forgiveness moderates the indirect path of parental rejection to smartphone addiction through the sense of security, while interpersonal forgiveness moderates the direct path of parental rejection to smartphone addiction. However, when Model 17 was tested (see Table 2), neither interaction of parental rejection nor forgiveness were significantly related to insecurity and smartphone addiction (*β* = −0.06, *p >* 0.05; *β* = −0.05, *p* > 0.05). In this joint model, the interaction terms were no longer significant, likely due to reduced statistical power from estimating multiple interactions simultaneously with a modest effect size.

**Figure 4:**
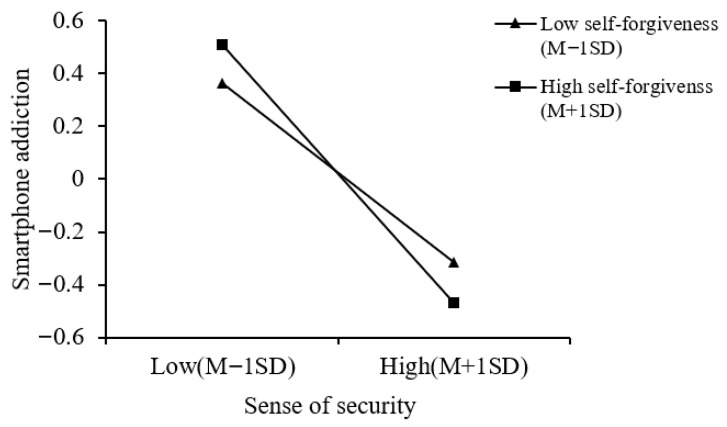
The moderating role of self-forgiveness in the relationship between sense of security and smartphone addiction (parental rejection Model 15).

A simple slope analysis of the moderation effect of self-forgiveness on the relationship between sense of security and smartphone addiction (see Figure 4) revealed that, regardless of whether forgiveness was at a high (*simple slope* = −0.49, *p* < 0.001) or low level (*simple slope* = −0.34, *p* < 0.001), sense of security was significantly related to adolescents’ smartphone addiction. However, when self-forgiveness was at a high level, the negative association between sense of security and smartphone addiction was significantly stronger. This suggested that as the level of self-forgiveness increases, the negative association between sense of security and smartphone addiction gradually intensifies.

**Figure 5:**
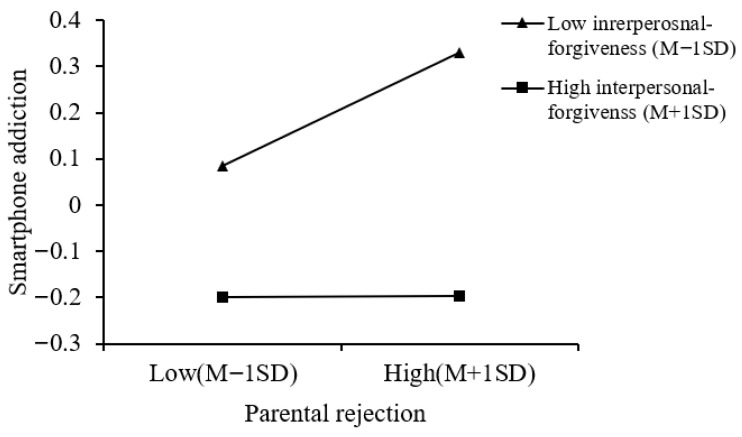
The moderating role of interpersonal forgiveness in the relationship between parental rejection and smartphone addiction (parental rejection Model 15).

Moreover, a simple slope analysis of the moderation effect of interpersonal forgiveness on the relationship between parental rejection and smartphone addiction (see Figure 5) showed that, at high levels of interpersonal forgiveness, parental rejection was not significantly related to adolescent smartphone addiction (*simple slope* = 0.001, *p* > 0.05). However, at low levels of interpersonal forgiveness, parental rejection was significantly related to adolescent smartphone addiction (*simple slope* = 0.13, *p* < 0.01). This suggests that the link of parental rejection and smartphone addiction is stronger for those with low levels of interpersonal forgiveness.

### 4.4. Moderated Mediation Effect of Parental Overprotection on Smartphone Addiction

The results of Model 15 (see Tables S3 and S4) showed that, after controlling for gender and age, parental overprotection was negatively related to sense of security (*β_SF_* = −0.34, *p* < 0.001; *β_IF_* = −0.34, *p* < 0.001), and sense of security was negatively related to smartphone addiction (*β_SF_* = −0.41, *p* < 0.001; *β_IF_* = −0.34, *p* < 0.001). Therefore, sense of security partially mediates the relationship between parental overprotection and smartphone addiction, with a mediation effect of 0.136 and a 95% CI of [0.100, 0.176], accounting for 47.92% of the total effect. In the separate model test, the interaction of parental overprotection and interpersonal forgiveness, and the interaction of sense of security and self-forgiveness were related to smartphone addiction (*β* = −0.07, *p* < 0.05; *β* = −0.07, *p* < 0.05. These findings suggest that interpersonal forgiveness moderates the direct pathway of parental overprotection directly affecting smartphone addiction, and self-forgiveness moderates the indirect pathway of parental overprotection affecting smartphone addiction through a sense of security. In the joint model (see Table 3), the interaction of parental overprotection and interpersonal forgiveness was negatively related to smartphone addiction (*β* = −0.07, *p* < 0.05), but the interaction of sense of security and self-forgiveness was failed to significantly related to smartphone addiction (*β* = −0.05, *p* > 0.05), which is likely due to reduced statistical power from estimating multiple interactions simultaneously.

**Figure 6:**
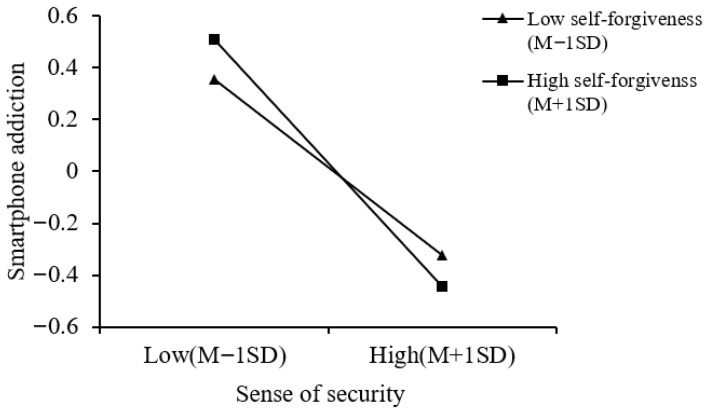
The moderating role of self-forgiveness in the relationship between sense of security and smartphone addiction (parental overprotection Model 15).

A simple slope analysis of the moderation effect of self-forgiveness on the relationship between sense of security and smartphone addiction (see Figure 6) revealed that, regardless of whether forgiveness was at a high (*simple slope* = −0.48, *p* < 0.001) or low level (*simple slope* = −0.34, *p* < 0.001), sense of security was significantly related to adolescent smartphone addiction. However, when self-forgiveness was at a high level, the relationship between sense of security and smartphone addiction was significantly stronger. This suggests that as the level of self-forgiveness increases, the negative association between sense of security and smartphone addiction gradually intensified.

**Figure 7:**
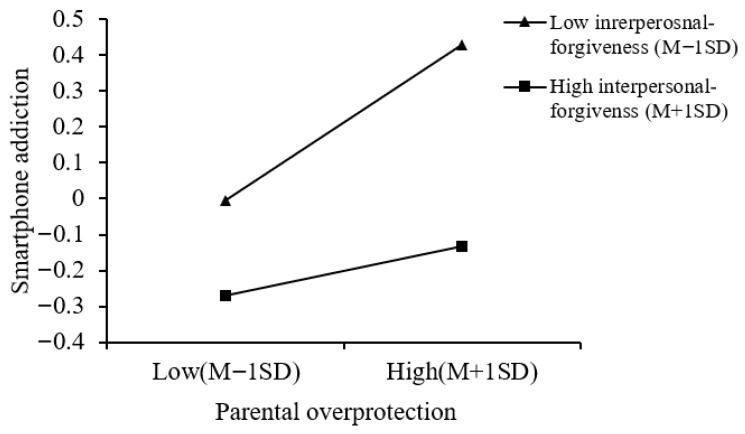
The moderating role of interpersonal forgiveness in the relationship between parental rejection and smartphone addiction (parental overprotection Model 15).

Moreover, a simple slope analysis of the moderation effect of interpersonal forgiveness on the relationship between parental overprotection and smartphone addiction (see Figure 7) revealed that, at high levels of forgiveness, the link of parental overprotection and adolescent smartphone addiction was not significant (*simple slope* = 0.07, *p* > 0.05). However, at low levels of interpersonal forgiveness, the link of parental overprotection and smartphone addiction was significant (*simple slope* = 0.22, *p* < 0.001). This suggests that the association between parental overprotection and smartphone addiction is stronger for those adolescents with low levels of interpersonal forgiveness.

## 5. Discussion

This study explored two critical questions regarding how (sense of security) and when (forgiveness) parental rejection and overprotection are related to smartphone adolescents’ addiction. These findings have significant theoretical implications for deepening and expanding our understanding of the relationship between parenting and adolescent smartphone addiction. Additionally, they offer new insights into prevention and intervention strategies for adolescent smartphone addiction.

### 5.1. The Relationships Between Parental Rejection, Overprotection and Smartphone Addiction

Research indicates that both parental rejection and overprotection are positively related to adolescent smartphone addiction, supporting hypotheses H1a and H1b. These findings are consistent with previous studies (Chen et al., 2021; He et al., 2024; Kong et al., 2025). However, this study expands upon previous research in at least two ways. First, the sample in this study includes adolescents in early and middle stages of development, providing a broader context for understanding the link between negative parenting and smartphone addiction. Second, this study is one of the first to examine parental rejection and overprotection concurrently among children, thereby providing a more comprehensive understanding of how these two parenting styles are associated with adolescent smartphone addiction.

Moreover, the findings provide empirical support for the resource conservation theory (Hobfoll, 2002), which suggests that when parents adopt rejecting or overprotective parenting styles, the amount of help, encouragement, and affirmation they provide to their children decreases, reducing the psychological resources available to the child (Fan et al., 2018). When children’s psychological needs are unmet in real life, they turn to the internet as an alternative source of fulfillment. Their possible over-reliance on this “virtual” psychological satisfaction leads to smartphone addiction (D. Li et al., 2016). Furthermore, parental overprotection undermines adolescents’ autonomy, reducing their motivation for self-exploration and breakthroughs, making them more likely to stay in their “comfort zone.” The immediate reinforcement and spatiotemporal flexibility provided by smartphones cater well to adolescents’ psychological characteristics, which further contributes to their addiction to smartphone use. These findings suggest that both parental rejection and overprotection are risk factors for adolescent smartphone addiction. They also imply that parents should provide more emotional warmth and behavioral support, respect their children’s autonomy, meet their psychological needs in a scientific and reasonable manner, and guide them in cultivating healthy habits in smartphone use.

### 5.2. The Mediating Role of Sense of Security in the Relationships of Parental Rejection, Overprotection and Smartphone Addiction

This study found that sense of security mediated the associations of both parental rejection and overprotection with adolescent smartphone addiction, suggesting that negative parenting practices may diminish adolescents’ security, which in turn is associated with higher addiction risk. This pattern is consistent with previous research linking negative parenting to lower emotional security (Stronach et al., 2013). However, the mechanisms through which parental rejection and overprotection undermine security may differ. In the case of parental rejection, the core dynamic is emotional distance. Rejecting parents provide little warmth or involvement, making it difficult for adolescents to develop close, trusting relationships with them. As a result, adolescents struggle to form secure attachments, leaving their need for emotional safety unfulfilled. In the case of parental overprotection, the core dynamic is the suppression of autonomy. While parental overprotection may satisfy adolescents’ material needs, it harms their autonomy (Jiang et al., 2010) and overlooks their genuine psychological needs. Particularly during adolescence, overprotection either causes the child to lose the sense of autonomy or triggers stronger rebellion, making it difficult for them to develop a secure attachment to their parents. Both pathways—emotional distance from rejection and autonomy suppression from overprotection—may have underlying neurocognitive and psychophysiological correlates. As Christou and Bacopoulou (2025) suggest, parent–child emotional dynamics are grounded in biobehavioral pathways that shape the development of emotion regulation circuits. Future research could examine whether these mechanisms mediate the link between negative parenting and adolescent insecurity.

On the other hand, sense of security was negatively related to adolescent smartphone addiction. Insecure parent–child attachment reduces adolescents’ experiences of emotional warmth and social support (Sun et al., 2018), leading to unmet psychological needs in everyday parent–child interactions. According to the use and gratification theory, individuals may use smartphones to satisfy their psychological needs (Kang & Jung, 2014), such as security and interpersonal connection. When an individual’s sense of security is fulfilled through smartphone use, they tend to use their phones more frequently, ultimately leading to addiction. Furthermore, according to phone attachment theory (J. H. Kim & Koh, 2018), adolescents may develop an emotional attachment to their phones similar to human attachment, which also contributes to smartphone addiction. These findings firstly suggest that the sense of security plays a crucial role in buffering the adverse effects of negative parenting on mobile phone addiction. Therefore, parents should actively cultivate a close parent–adolescent relationship to enhance adolescents’ sense of security. Meanwhile, adolescents should strive to maintain a positive parent–child bond and engage in responsible and balanced smartphone use to reduce the risk of smartphone addiction.

### 5.3. The Moderating Role of Forgiveness in the Relationships of Parental Rejection, Overprotection and Smartphone Addiction

The results regarding the moderating effect of forgiveness diverged between the separate and joint models. Our theoretical framework specifically hypothesized that interpersonal forgiveness and self-forgiveness operate on different paths. Therefore, the separate models directly test these theory-driven predictions. The saturated model, which simultaneously estimates four interaction terms, serves as a more conservative test. However, detecting two simultaneous interaction effects of small magnitude (β = 0.05–0.07, as observed in our separate models) requires substantially larger statistical power. Post hoc power analysis indicated that detecting an effect of this size with 80% power would require over 1500 participants for a model with four interaction terms. Given our sample size (N = 677), the lack of significance in the saturated model is likely attributable to reduced statistical power rather than the absence of true effects. Thus, we interpret the separate model results as the primary test of our hypotheses, while the saturated model provides a useful but underpowered robustness check.

The results showed that self-forgiveness moderated the relationship between sense of security and smartphone addiction. In particular, the negative effect is more significant for the individuals of high self-forgiveness. For those with low self-forgiveness, their sense of security is more based on the maintenance of a sense of control over the environment, which is effective but has a relatively limited protective effect; additionally, it relies on the stability of external conditions and is easily eroded when faced with the threat of loss of control. For those with high self-forgiveness, they have psychological resources for self-acceptance in addition to a sense of security. Self-forgiveness allows them to forgive their shortcomings and accept imperfect realities, and this internal stability makes the existing sense of security more protective. This also confirms the view of the protective factor enhancement model (Zimmerman et al., 2013), where forgiveness and security play a synergistic protective role.

Meanwhile, interpersonal forgiveness moderates the direct pathways between parental rejection, overprotection, and adolescent smartphone addiction. As the tendency for interpersonal forgiveness increases, the negative effects of parental rejection and overprotection on adolescent smartphone addiction decrease. Compared with self-forgiveness, the moderating role of interpersonal forgiveness was particularly robust. Even in a saturated model (Model 17) that simultaneously controlled for the effects of self-forgiveness and all other interaction terms, the protective effect of interpersonal forgiveness against parental overprotection remained statistically significant. This finding underscores the unique role that forgiving others plays in helping adolescents cope with the resentment and anger triggered by overly intrusive parenting. Parental rejection may trigger negative emotions such as resentment and dissatisfaction in adolescents, and interpersonal forgiveness can serve as an important coping mechanism for these emotions. Freedman and Knupp (2003) highlighted that forgiveness is an effective way to cope with negative emotions (e.g., resentment, dissatisfaction), especially interpersonal forgiveness, which can help alleviate the negative emotions caused by parental rejection. This also aligns with the stress-buffering hypothesis (Cohen & Wills, 1985), which suggests that individuals’ positive traits can effectively mitigate the potential negative impact of stress on psychological functioning. Therefore, interpersonal forgiveness seems to play a crucial role in repairing the relationship between adolescents raised in a parental rejection environment and their parents, ultimately reducing the impact of negative emotions, a key risk factor for excessive smartphone use. In addition, moderation analysis shows that interpersonal forgiveness effectively reduces the effect of parental overprotection on adolescent smartphone addiction, especially for adolescents with high levels of interpersonal forgiveness. As mentioned above, parental overprotection diminishes adolescents’ autonomy (Jiang et al., 2010). Meanwhile, adolescents’ rebellious tendencies during puberty further amplify the intensity of unmet psychological needs. According to the compensatory internet use theory (Kardefelt-Winther, 2014), this unmet need for autonomy drives adolescents to seek psychological “compensation” in smartphones, leading to excessive smartphone use. Interpersonal forgiveness, by helping adolescents relinquish negative emotions toward their parents, reduces the psychological need to seek compensatory satisfaction from smartphones.

The current study is the first to clarify the conditions under which parental rejection and overprotection have a strong association with smartphone addiction and to reveal the role of forgiveness between them. While the effect size of the moderating effect is relatively small for both interpersonal forgiveness and self-forgiveness, it still has statistical significance. These findings suggest that interpersonal and self-forgiveness serve different protective functions against smartphone addiction in adolescents. Enhancing adolescents’ capacity for forgiveness may effectively mitigate the negative impact of maladaptive parenting practices.

### 5.4. Limitations and Future Directions

This study was among the first to examine the relationship between parental rejection, overprotection and adolescent smartphone addiction, and it provided preliminary insights into the important roles of sense of security and forgiveness between them. However, there are several limitations in this study.

First, causal relationships between parenting behaviors and smartphone addiction cannot be established due to the cross-sectional nature of the study design. Although we discuss potential directional pathways (e.g., negative parenting leading to smartphone addiction) based on theoretical frameworks, alternative directions (e.g., smartphone addiction eliciting more negative parenting) are equally plausible and should be examined in future longitudinal research. Additionally, adolescence encompasses distinct developmental stages, yet the present study only recruited participants in early and middle adolescence. Future research should include the unified stage of adolescence to examine the full trajectory of the effects of parenting behaviors on smartphone addiction.

Second, the study relied on self-report methods from adolescents, which may be subject to social desirability bias and recall errors and also introduce common method bias and inflate the observed associations. While Harman’s single-factor test suggested that this is not a major concern, this test is not definitive. Future studies could employ more objective and multi-source data collection methods (such as multi-object reporting methods) to provide a more accurate depiction of the relationships between variables.

Third, although mean imputation was used for its simplicity given the low missing rate, we acknowledge that it may reduce variability. Future studies with larger datasets could employ more sophisticated techniques like multiple imputation.

Finally, parental parenting may involve both positive and negative parenting practices, and smartphone addiction itself includes specific behaviors (e.g., gaming, short videos). Future research could further refine the variables and explore the relationships between more specific factors in greater depth.

## 6. Conclusions

(1) Parental rejection and overprotection are positively related to adolescent smartphone addiction.
(2) Sense of security mediates the relationships between parental rejection, overprotection, and adolescent smartphone addiction.
(3) Interpersonal forgiveness moderates the relationships between parental rejection, overprotection, and adolescent smartphone addiction, and self-forgiveness moderates the relationships between sense of security and smartphone addiction.

## Figures and Tables

**Table 1:** Descriptive statistics and correlation analysis.

	M (SD)	1	2	3	4	5	6	7	8	9
1 Sex	-	-								
2 Age	12.15 (1.31)	−0.10 **	-							
3 Parental rejection	16.71 (5.62)	−0.04	0.06	-						
4 Parental overprotection	33.10 (6.84)	−0.11 **	−0.07	0.40 ***	-					
5 Sense of security	53.65 (12.30)	−0.12 **	−0.09 *	−0.36 ***	−0.31 ***	-				
6 Smartphone addiction	43.47 (14.91)	−0.07	0.21 ***	0.28 ***	0.27 ***	−0.46 ***	-			
7 Forgiveness	9.94 (15.01)	−0.05	−0.04	−0.26 ***	−0.19 ***	0.41 ***	−0.34 ***	-		
8 Self-forgiveness	1.14 (7.54)	−0.11 **	0.01	−0.16 **	−0.20 **	0.40 **	−0.18 **	0.68 **	-	
9 Interpersonal forgiveness	8.80 (11.28)	0.01	0.06	−0.25 **	−0.12 **	0.28 **	−0.33 **	0.87 **	0.24 **	-

**Note:** N = 677; Gender was dummy coded, with males coded as 1 and females coded as −1; M (SD) = Mean (Standard Deviation); Dashes (“-”) on the diagonal represent the correlation of a variable with itself, which is constant (1) and therefore omitted; *** *p* < 0.001, ** *p* < 0.01, * *p* < 0.05.

**Table 2:** The moderated mediation effect of forgiveness between parental rejection and smartphone addiction (Model 17).

		Fitness Indexes			Coefficients Significance			
		*R*	*R* ^2^	*F*	*Β*	*T*	LLCI	ULCI
Sense of security	Parental rejection	0.40	0.16	41.40 ***	−0.37	−10.27 ***	−0.434	−0.295
	Sex				−0.28	−3.88 ***	−0.416	−0.136
	Age				−0.07	−2.29 *	−0.134	−0.010
Smartphone addiction	Parental rejection	0.55	0.30	28.88 ***	0.06	1.55	−0.016	0.136
	Sense of security				−0.38	−9.94 ***	−0.460	−0.308
	Interpersonal forgiveness (IF)				−0.20	−5.49 ***	−0.265	−0.125
	Self-forgiveness (SF)				0.03	0.80	−0.042	0.099
	Parental rejection × IF				−0.06	−1.67	−0.122	0.010
	Parental rejection × SF				−0.03	−0.9	−0.101	0.036
	Sense of security × IF				−0.04	−1.15	−0.110	0.029
	Sense of security × SF				−0.05	−1.60	−0.115	0.012
	Sex				−0.18	−2.78 **	−0.314	−0.054
	Age				0.14	4.65 ***	0.078	0.192

**Note:** LLCI and ULCI represent the lower and upper bounds of the 95% confidence interval estimated using the bootstrap method, respectively. *** *p* < 0.001, ** *p* < 0.01, * *p* < 0.05.

**Table 3:** The moderated mediation effect of forgiveness between parental overprotection and smartphone addiction (Model 17).

		Fitness Indexes			Coefficients Significance			
		*R*	*R* ^2^	*F*	*β*	*t*	LLCI	ULCI
Sense of security	Parental overprotection	0.37	0.14	35.30 ***	−0.34	−9.36 ***	−0.409	−0.267
	Sex				−0.33	−4.54 ***	−0.471	−0.187
	Age				−0.12	−3.62 **	−0.179	−0.053
Smartphone addiction	Parental overprotection	0.56	0.31	30.44 ***	0.15	4.31 ***	0.081	0.218
	Sense of security				−0.36	−9.52 ***	−0.433	−0.285
	Interpersonal forgiveness (IF)				−0.21	−5.86 ***	−0.273	−0.136
	Self-forgiveness (SF)				0.03	0.94	−0.037	0.105
	Parental overprotection × IF				−0.07	−1.97 *	−0.139	0.000
	Parental overprotection × SF				−0.01	−0.14	−0.074	0.064
	Sense of security × IF				−0.04	−1.04	−0.102	0.032
	Sense of security × SF				−0.05	−1.50	−0.113	0.015
	Sex				−0.15	−2.25 *	−0.280	−0.019
	Age				0.15	5.08 ***	0.091	0.205

**Note:** LLCI and ULCI represent the lower and upper bounds of the 95% confidence interval estimated using the bootstrap method, respectively. *** *p* < 0.001, ** *p* < 0.01, * *p* < 0.05.

## Data Availability

The datasets generated and analyzed during the current study are not publicly available but are available from the corresponding author on reasonable request.

## Supplementary Materials

The following supporting information can be downloaded at https://www.mdpi.com/article/10.3390/bs16050796/s1. Supplementary Table S1. The moderated mediation effect of self-forgiveness between parental rejection and smartphone addiction (model 15). Supplementary Table S2. The moderated mediation effect of interpersonal-forgiveness between parental rejection and smartphone addiction (model 15). Supplementary Table S3. The moderated mediation effect of self-forgiveness between parental overprotection and smartphone addiction (model 15). Supplementary Table S4. The moderated mediation effect of interpersonal-forgiveness between parental overprotection and smartphone addiction (model 15).
