# Supplementary material for: Parental Rejection, Overprotection and Adolescent Smartphone Addiction: Mediating Role of Sense of Security and Moderating Role of Forgiveness
Source: Behav Sci (Basel). 2026 May 16;16(5):796. doi: 10.3390/bs16050796 (PMC13203291; doi:10.3390/bs16050796)
Supplement: Supplementary file 1 [file behavsci-16-00796-s001.zip › behavsci-4278630-supplementary.pdf]

Supplementary Table S1. The moderated mediation effect of self-forgiveness between parental rejection and smartphone addiction(model15).

		Fitness indexes			Coefficients significance			
		R	R^2	*F*	*B*	*T*	LLCI	ULCI
Sense of security	Parental rejection	0.40	0.16	41.40***	-0.37	-10.27***	-0.434	-0.295
	Sex				-0.28	-3.88***	-0.416	-0.136
	Age				-0.07	-2.29*	-0.134	-0.010
Smartphone addiction	Parental rejection	0.52	0.27	34.83***	0.11	2.80**	0.032	0.182
	Sense of security				-0.41	-10.6***	-0.490	-0.337
	Self-forgiveness(SF)				-0.01	-0.08	-0.074	0.068
	Parental rejection×SF				-0.05	-1.13	-0.112	0.022
	Sense of security×SF				-0.08	-2.41*	-0.137	-0.014
	Sex				-0.20	-2.90**	-0.329	-0.064
	Age				0.14	4.75***	0.083	0.199

Note: LLCI and ULCI represent the lower and upper bounds of the 95% confidence interval estimated using the bootstrap method, respectively.****p* < .001, ***p* < .01, **p* < .05.

Supplementary Table S2. The moderated mediation effect of interpersonal-forgiveness between parental rejection and smartphone addiction(model15).

		Fitness indexes			Coefficients significance			
		R	R^2	*F*	*B*	*T*	LLCI	ULCI
Sense of security	Parental rejection	0.40	0.16	41.40***	-0.37	-10.27***	-0.434	-0.295
	Sex				-0.28	-3.88***	-0.416	-0.136
	Age				-0.07	-2.29*	-0.134	-0.010
Smartphone addiction	Parental rejection	0.55	0.30	40.77***	0.07	1.80	-0.006	0.140
	Sense of security				-0.37	-10.17***	-0.439	-0.297
	interpersonal-forgiveness(IF)				-0.20	-5.67***	-0.265	-0.129
	Parental rejection×IF				-0.07	-2.05*	-0.129	-0.003
	Sense of security×IF				-0.06	-1.68	-0.121	0.009
	Sex				-0.19	-2.85**	-0.317	-0.058
	Age				0.14	4.66***	0.078	0.192

Note: LLCI and ULCI represent the lower and upper bounds of the 95% confidence interval estimated using the bootstrap method, respectively.****p* < .001, ***p* < .01, **p* < .05.

Supplementary Table S3. The moderated mediation effect of self-forgiveness between parental overprotection and smartphone addiction(model15).

		Fitness indexes			Coefficients significance			
		R	R^2	F	B	T	LLCI	ULCI
Sense of security	Parental overprotection	0.37	0.14	35.30***	-0.34	-9.36***	-0.409	-0.267
	Sex				-0.33	-4.54***	-0.471	-0.187
	Age				-0.12	-3.62**	-0.179	-0.053
Smartphone addiction	Parental overprotection	0.52	0.27	35.78***	0.15	4.29***	0.083	0.223
	Sense of security				-0.41	-10.73***	-0.481	-0.332
	self-forgiveness(SF)				-0.01	0.21	-0.064	-0.079
	Parental overprotection×SF				-0.01	-0.40	-0.080	-0.053
	Sense of security×SF				-0.07	-2.19*	-0.131	0.007
	Sex				-0.16	-2.36*	-0.294	-0.027
	Age				0.16	5.36***	0.101	0.218

Note: LLCI and ULCI represent the lower and upper bounds of the 95% confidence interval estimated using the bootstrap method, respectively.***p < .001, **p < .01, *p < .05.

Supplementary Table S4. The moderated mediation effect of interpersonal-forgiveness between parental overprotection and smartphone addiction(model15).

		Fitness indexes			Coefficients significance			
		R	R^2	F	B	T	LLCI	ULCI
Sense of security	Parental overprotection	0.37	0.14	35.30***	-0.34	-9.36***	-0.409	-0.267
	Sex				-0.33	-4.54***	-0.471	-0.187
	Age				-0.12	-3.62**	-0.179	-0.053
Smartphone addiction	Parental overprotection	0.56	0.31	43.00***	0.14	4.17***	0.076	0.211
	Sense of security				-0.34	-9.63***	-0.415	-0.274
	interpersonal-forgiveness(IF)				-0.21	-6.08***	-0.274	-0.140
	Parental overprotection×IF				-0.07	-2.23*	-0.139	-0.009
	Sense of security×IF				-0.05	-1.60	-0.114	0.012
	Sex				-0.16	-2.42*	-0.290	-0.030
	Age				0.15	5.04***	0.089	0.203

Note: LLCI and ULCI represent the lower and upper bounds of the 95% confidence interval estimated using the bootstrap method, respectively.***p < .001, **p < .01, *p < .05.
